# Opportunistic CT-derived analysis of fat and muscle tissue composition predicts mortality in patients with cardiogenic shock

**DOI:** 10.1038/s41598-023-49454-x

**Published:** 2023-12-15

**Authors:** Babak Salam, Muntadher Al Zaidi, Alois M. Sprinkart, Sebastian Nowak, Maike Theis, Daniel Kuetting, Adem Aksoy, Georg Nickenig, Ulrike Attenberger, Sebastian Zimmer, Julian A. Luetkens

**Affiliations:** 1 Department of Diagnostic and Interventional Radiology, University Hospital Bonn, Venusberg-Campus 1, 53127 Bonn, Germany; 2 Quantitative Imaging Lab Bonn (QILaB), Bonn, Germany; 3 Department of Internal Medicine II, Heart Center Bonn, University Hospital Bonn, Venusberg-Campus 1, 53127 Bonn, Germany

**Keywords:** Predictive markers, Cardiovascular diseases, Outcomes research

## Abstract

Prognosis estimation in patients with cardiogenic shock (CS) is important to guide clinical decision making. Aim of this study was to investigate the predictive value of opportunistic CT-derived body composition analysis in CS patients. Amount and density of fat and muscle tissue of 152 CS patients were quantified from single-slice CT images at the level of the intervertebral disc space L3/L4. Multivariable Cox regression and Kaplan–Meier survival analyses were performed to evaluate the predictive value of opportunistically CT-derived body composition parameters on the primary endpoint of 30-day mortality. Within the 30-day follow-up, 90/152 (59.2%) patients died. On multivariable analyses, lactate (Hazard Ratio 1.10 [95% Confidence Interval 1.04–1.17]; *p* = 0.002) and patient age (HR 1.04 [95% CI 1.01–1.07], *p* = 0.017) as clinical prognosticators, as well as visceral adipose tissue (VAT) area (HR 1.004 [95% CI 1.002–1.007]; *p* = 0.001) and skeletal muscle (SM) area (HR 0.987 [95% CI 0.975–0.999]; *p* = 0.043) as imaging biomarkers remained as independent predictors of 30-day mortality. Kaplan–Meier survival analyses showed significantly increased 30-day mortality in patients with higher VAT area (*p* = 0.015) and lower SM area (*p* = 0.035). CT-derived VAT and SM area are independent predictors of dismal outcomes in CS patients and have the potential to emerge as new imaging biomarkers available from routine diagnostic CT.

## Introduction

Despite novel treatment options, cardiogenic shock (CS) remains a condition associated with a high mortality rate and uncertain prognosis^1^. Universal models for predicting outcomes and, more importantly, guiding optimal treatment decisions remain elusive^2^. Age is a known, non-modifiable risk factor for mortality in patients with CS and the most prevalent patient-related risk factor among published CS prognostic scores^2,3^.

Overall, aging is characterized by a chronic, low-grade proinflammatory state leading to greater vulnerability to multimorbidity, disability, and death (“inflammaging”)^4^. In this regard, adipose tissue, as the largest endocrine organ in humans, fulfills a crucial role in age-related metabolic disorders and lifespan^5^. Age-related inflammatory changes in adipose tissue are manifested, among other mechanisms, by a shift in adipose tissue distribution from subcutaneous to visceral fat depots^6^. Subcutaneous abdominal adipose tissue (SAT) and visceral abdominal adipose tissue (VAT) have both been reported to be associated with increased morbidity, with a stronger association described for VAT^7,8^. VAT plays an important role in the pathophysiology of cardiometabolic disease by increasingly secreting adipokines, which consecutively leads to a subclinical inflammatory state^6,7^. While chronic low-grade inflammation develops with aging, it is also evident that age-related inflammatory conditions exacerbate the aging process. Multimorbidity can cause an accumulation of deficits participating in frailty onset and evaluation. Within this scenario, inflammaging is directly correlated to an increased risk of age-related chronic diseases and frailty^9^. Sarcopenia as part of frailty syndrome was shown to be associated with cardiovascular disease and worsened outcomes following myocardial infarction^10,11^. Moreover, previous studies indicated that beyond the mere muscle mass itself, the fatty muscle fraction (FMF) as an indicator of muscle quality seems to be of prognostic value in several severe diseases, for instance in patients receiving mechanical circulatory support by veno-venous extracorporeal membrane oxygenation^12^.

One way to accurately quantify changes in body composition in the context of inflammaging and frailty is offered by computed tomography. In the past, computed tomography (CT)-derived parameters of body composition have shown predictive value for numerous cardiovascular diseases and malignancies^7,8,13,14^. Although factors such as changes in hemodynamics complicate the acquisition of CT in patients with cardiogenic shock, there are several clinical scenarios in which CT examinations are essential in these patients^15,16^.

In this study we aimed to investigate the extent to which CT-derived parameters of fat and muscle composition as surrogates for the clinical status are related to adverse outcome in patients with cardiogenic shock.

## Methods

### Study population

**Figure 1:**
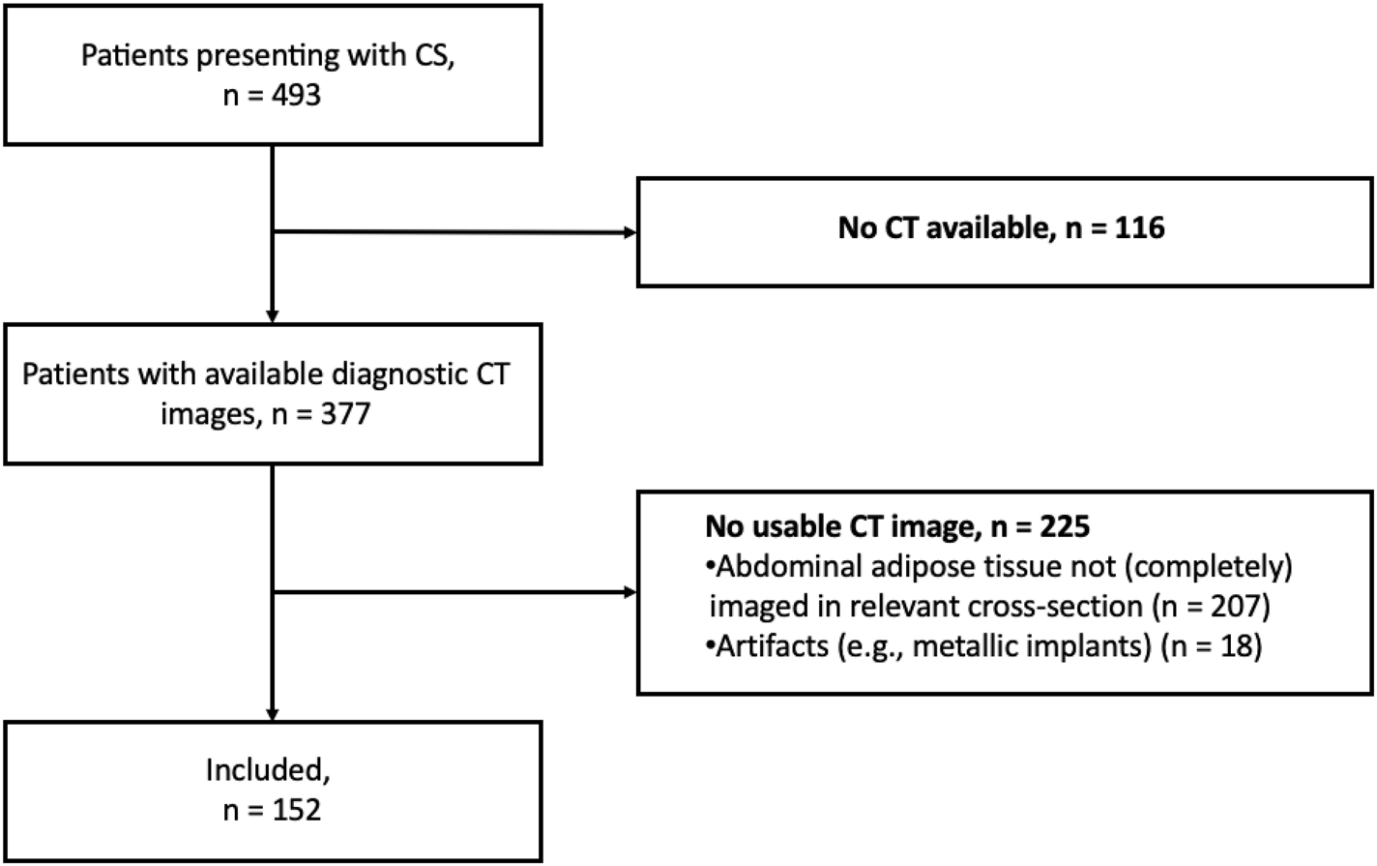
Flow diagram shows included patients presenting with cardiogenic shock (CS) and patients who had to be excluded from analysis.

The study was approved by the Ethics Committee of the University of Bonn (Medical Faculty) and the need for written informed consent was waived due to the retrospective monocentric study design. The study was carried out in compliance with the ethical standard set in the 1964 Declaration of Helsinki as well as its later amendments. A total of 493 patients who presented with cardiogenic shock (CS) between March 2019 and December 2021 at the University Hospital Bonn were initially retrospectively evaluated. CS was defined as a “primary cardiac dysfunction resulting in an inadequate cardiac output”, according to current heart failure guidelines of the European society of cardiology^17^. Patients who had diagnostic CT scans within 1 week of hospitalization were identified. In these patients, the intervertebral disc space L3/L4 needed to be covered by the scan range as this level served as the anatomical landmark for conduction of body composition measurements. After exclusion of patients without CT or with images with severe artifacts, a total of 152 patients were included for final analysis (see study flowchart Fig. 1). Medical records were reviewed to retrieve clinical variables and baseline physical characteristics of included patients. The primary endpoint of this study was all-cause mortality after 30 days.

### Image analysis

Tissue areas measured from axial single-slice cross-sectional images at the level of the intervertebral disc space L3/L4 were previously demonstrated to be highly correlated with compartment volumes of adipose tissues (visceral adipose tissue, VAT; subcutaneous adipose tissue, SAT) and skeletal muscles (SM) and hence were used for image analysis^18^. For each patient, pseudoanonymized single-slice images at this level were retrieved from the local picture archiving and communication system (IMPAX, Dedalus HealthCare GmbH, Germany). Image analysis was performed using an end-to-end automated deep learning pipeline for body composition analysis from abdominal CT scans^19^. Only arterial phase scans were considered for body composition analysis, to prevent any potential bias due to the contrast phase. The area was obtained for each compartment (SAT, VAT, SM). In addition to the area of the individual compartments, the mean densities of SAT and VAT were determined. In inflammatory active adipose tissue, local adipogenesis is inhibited by inflammatory mediators, resulting in a shift of the density values from the lipid phase to the aqueous phase^20^. Mean adipose tissue density can therefore be considered as a surrogate for inflammatory activity of adipose tissue in the context of inflammaging.

**Figure 2:**
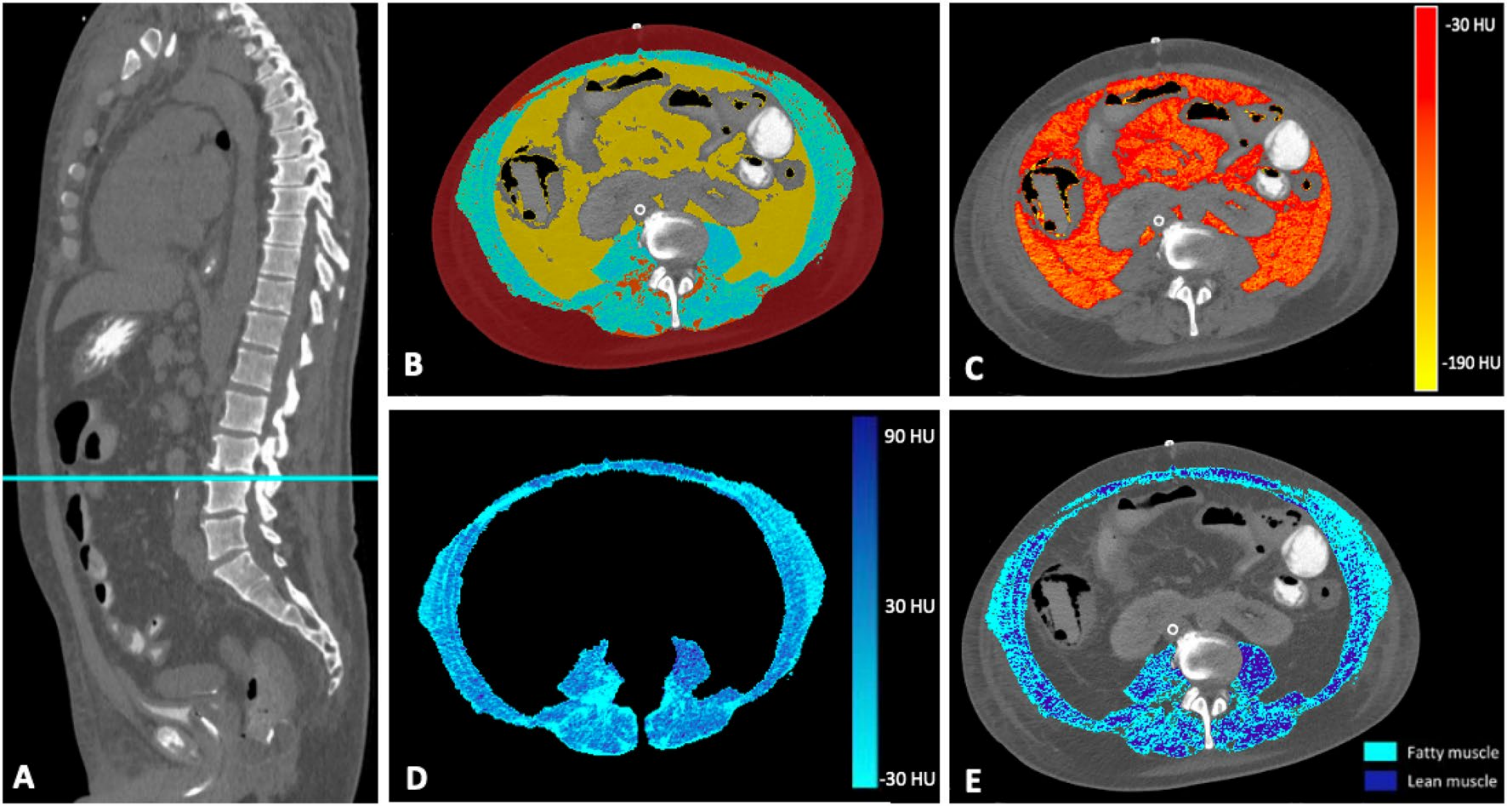
Synopsis of body composition analysis. Single-slice cross sectional CT images at the level of the intervertebral disc space L3/L4 were exported for analysis (**A**). Areas of visceral adipose tissue (yellow) were separated from subcutaneous adipose tissue (red) and abdominal skeletal muscle (blue) and assessed within these cross-sections based on densitometric thresholds (**B**). In addition to adipose tissue area, mean attenuation of visceral adipose tissue area was calculated to determine overall inflammatory activity (**C**). Mean muscle attenuation of the entire skeletal muscle area was calculated to obtain overall myosteatosis (**D**). Muscular fat infiltration was also quantified by separating the skeletal muscle area into areas of fatty and lean muscle using attenuation thresholds of − 30 to 29 Hounsfield units (HU) and 30 to 100 HU, respectively (**E**). CT = computed tomography, HU = Hounsfield Units.

Based on the automated segmentation results, further biomarkers for the analysis of myosteatosis were calculated. Myosteatosis as a measure of muscle quality in the context of frailty was shown to be associated with cardiovascular risk factors^21,22^. Myosteatosis is characterized by intra- and inter-myocellular deposition of triglycerides^21^. Two methods for determination of myosteatosis were therefore applied. First, the mean attenuation of the entire skeletal muscle area was calculated to obtain overall myosteatosis. Second, to quantify muscular fat infiltration more accurately, intra-myocellular deposition of triglycerides was quantified calculating fatty muscle fraction (FMF) as a proven objective surrogate for frailty^12,23^. Therefore, the paraspinal skeletal muscle area was separated into areas of fatty and lean muscle based on commonly accepted attenuation thresholds^21^. Accordingly, fatty and lean muscle were defined by attenuation threshold ranges of low (− 29 to 29 HU) and high muscle attenuation (30 to 100 HU), respectively (Fig. 2). FMF was then calculated as the area of low attenuation muscle tissue referred to the skeletal muscle area^12^. For calculation of inter-myocellular fat deposition within paraspinal skeletal muscle area, pixels within the defined attenuation threshold range (− 190 to − 30 HU) were counted and multiplied with the respective pixel surface area^23^. Intermuscular fat fraction was calculated as the proportion of adipose tissue in paraspinal skeletal muscle tissue.

### Statistical analysis

SPSS Statistics 28 (IBM, Armonk, NY, USA) was used for statistical analysis. Patient characteristics are presented as mean ± standard deviation (SD) for normally distributed variables, as median (interquartile range) for non-normally distributed variables and as counts and percentages for categorical variables. Continuous variables were checked for normal distribution. Readily obtainable clinical and laboratory data (patient age, lactate, creatinine) with demonstrated predictive value^2^ were analyzed alongside with body composition metrics with potential impact on survival by univariable and subsequent multivariable Cox regression analyses. Results are displayed as adjusted hazard ratio (HR) with 95% confidence interval (CI). With continuous variables, the hazard ratio indicates the change in the risk of 30-day mortality, upon the rise of the parameter by one unit (e.g. rise of SM area by 1 cm^2^). The Kaplan–Meier method with log-rank tests was used to investigate the association between the VAT area, as well as the SM area and survival time. For this purpose, the lowest tertile of each group was compared with the two upper tertiles. Kaplan–Meier survival analyses were performed, and groups were compared by log-rank p-value calculation. The two-sample Student's t-test was used to compare continuous variables between the high VAT area group and the low VAT area group, and the high SM area group and the low SM area group, respectively. Dichotomous variables were compared using the χ2 test. Correlation analysis was performed by Pearson or Spearman correlation coefficients, depending on linearity of association. The level of statistical significance was set to *p* < 0.05.

## Results

### Clinical and anthropometric baseline characteristics of the study population

We included 152 patients with CS and applicable CT data (Fig. 1). Table 1 summarizes the baseline characteristics of the study population. The mean age was 68.6 ± 13.1 years and 72.4% (110/152) of the population were male. 66.4% (101/152) presented with cardiac arrest and etiology of CS was acute myocardial infarction in 50.7%. Median lactate at admission 6.4 (2.9–10.2) mmol/l and median creatinine was 1.6 (1.2–2.2) mg/dl. The incidence of the primary endpoint of 30-day mortality was 59.2% (90/152). Supplemental Table 1 presents the baseline characteristics stratified by 30-day survivors and non-survivors.

**Table 1:** Clinical, anthropometric and CT-derived baseline characteristics of the study population (n = 152) presenting with cardiogenic shock (CS).

Variables	Patient cohort (n = 152)
Age (years)	68.6 ± 13.1
Male sex	110 (72.4%)
Acute myocardial infarction	77 (50.7%)
Cardiac arrest (%)	101 (66.4%)
Lactate (mmol/l)	6.4 (2.9–10.2)
pH	7.26 (7.10–7.37)
Base excess	− 8.0 (− 12.5 to − 4.48)
Creatinine (mg/dl)	1.6 (1.2–2.2)
Hemoglobin (g/dl)	11.7 ± 2.7
White blood cell count (10^3^/µl)	13.9 (10.3–20.1)
Platelet count (10^3^/µl)	205 (149–302)
C-reactive protein (mg/l)	15.7 (5.0–85.1)
30-day mortality	90 (59.2%)
Median overall survival (days)	16.5 (4–30)
Skeletal muscle area (SM area, cm^2^)	153.8 ± 34.2
Fat tissue in muscle area (cm^2^)	20.9 ± 12.5
Visceral adipose tissue area (VAT area, cm^2^)	210.4 ± 127.3/199.6 (125.5–290.3)
Subcutaneous adipose tissue area (cm^2^)	235.9 ± 117.3/222.6 (160.6–293.7)

Data are presented as n (%), mean ± standard deviation if normally distributed and as median (interquartile-range) if not normally distributed. Laboratory values at admission are shown.

### Predictive value of CT derived cardiac fat parameters in a predictive model

To determine the prognostic significance of clinical, laboratory- and CT-derived parameters, univariable cox regression was performed (Table 2). Regarding demographic variables, patient age was positively associated with 30-day mortality (hazard ratio [95% confidence interval]: 1.04 (1.02–1.06), *p* < 0.001), whereas no significant association was observed for male sex (HR [95% CI] 2.02 [0.73–5.57]; *p* = 0.17). In a subanalysis, BMI also exhibited no significant associations with 30-day survival following cardiogenic shock (HR [95% CI] 1.02 [0.95–1.10]; *p* = 0.58). There was no difference regarding the etiology of the CS (CS caused by myocardial infarction: HR [95% CI] 0.88 [0.58–1.42], *p* = 0.53). When analyzing laboratory parameters, lactate was significantly associated with 30-day mortality (HR [95% CI] 1.07 [1.01–1.13], *p* = 0.024). There was a non-significant trend found for creatinine (HR [95% CI] 1.15 [0.99–1.33], *p* = 0.062). Other laboratory parameters (c-reactive protein, hemoglobin, white blood cell count or platelet count) showed no association with the primary endpoint. An association with the primary endpoint was also not shown for cardiovascular risk factors such as diabetes and arterial hypertension (Table 2).

**Table 2:** Predictors of 30-day mortality in patients presenting with cardiogenic shock (CS) determined by using Cox regression univariate analysis.

Variables	Hazard ratio (95% CI)	*p* value
Anthropometric baseline characteristics		
Age	**1.04 (1.02–1.06)**	**< 0.001**
Male sex	2.02 (0.73–5.57)	0.173
Body mass index	1.02 (0.95–1.10)	0.579
Clinical and laboratory data		
Acute myocardial infarction	0.88 (0.58–1.42)	0.527
Arterial Hypertension	1.22 (0.37–4.14)	0.740
Diabetes	1.23 (0.74–2.24)	0.372
Lactate (mmol/l)	**1.07 (1.01–1.13)**	**0.024**
Creatinine (mg/dl)	1.15 (0.99–1.33)	0.062
Hemoglobin (g/dl)	0.93 (0.85–1.01)	0.087
White blood cell count (10^3^/µl)	1.00 (0.97–1.03)	0.912
Platelet count (10^3^/µl)	1.00 (1.00–1.00)	0.470
C-reactive protein (mg/l)	1.00 (1.00–1.01)	0.122
CT derived parameters		
Fatty muscle fraction	1.69 (0.46–6.18)	0.428
Inter-muscle fat fraction	**13.84 (2.11–90.84)**	**0.006**
Skeletal muscle area (SM area)	**0.993 (0.987–0.999)**	**0.033**
Fat tissue in muscle area	**1.02 (1.00–1.04)**	**0.017**
Visceral adipose tissue area (VAT area)	**1.002 (1.001–1.004)**	**0.010**
Subcutaneous adipose tissue area	0.65 (0.25–1.70)	0.381
Total skeletal muscle—mean HU	0.99 (0.98–1.01)	0.904
Fat tissue in muscle—mean HU	0.98 (0.95–1.00)	0.096
Visceral adipose tissue—mean HU	1.00 (0.98–1.02)	0.963
Subcutaneous adipose tissue—mean HU	1.00 (0.99–1.01)	0.552

Univariable Cox regression analysis. Hazard ratios are presented with 95% confidence interval.

Significant values are in bold.

Regarding CT-derived parameters (Table 2), an increase in inter-muscle fat fraction (HR [95% CI] 13.84 [2.11–90.84], *p* = 0.006), area of fat tissue in muscle (HR [95% CI] 1.02 [1.00–1.04], *p* = 0.017) and VAT area (HR [95% CI] 1.002 [1.001–1.004], *p* = 0.010) were associated with a higher incidence of the primary endpoint, whereas higher SM area was associated with lower 30-day mortality (HR [95% CI] 0.993 [0.987–0.999], *p* = 0.033). Fatty muscle fraction (FMF), which was previously described to be a prognosticator of outcome in other patient cohorts^12^, was not associated with 30-day mortality in our CS cohort (HR [95% CI] 1.69 [0.46–6.18], *p* = 0.428). Mean densities of abdominal adipose and muscle tissue compartments were not associated with the primary endpoint.

Next, we conducted a multivariable cox regression analysis using clinically relevant and CT-derived variables (age, lactate, creatinine, FMF, inter-muscle fat fraction, SM, fat tissue area in muscle, VAT). Here, lactate (HR [95% CI] 1.10 [1.04–1.17], *p* = 0.002) and patient age (HR [95% CI] 1.04 [1.01–1.07], *p* = 0.017), as well as VAT area (HR [95% CI] 1.004 [1.002–1.007], *p* = 0.001) and SM area (HR [95% CI] 0.987 [0.975–0.999], *p* = 0.043) were identified as independent predictors of 30-day mortality (see Table 3).

**Table 3:** Variables currently used in clinical practice for predicting patient outcome or showing an association with outcome on univariable analysis were included in a multivariable model and analyzed by multivariable Cox regression.

Variables	Hazard ratio (95% CI)	*p* value
Age	**1.04 (1.01–1.07)**	**0.017**
Lactate	**1.10 (1.04–1.17)**	**0.002**
Creatinine	1.09 (0.90–1.32)	0.382
Fatty muscle fraction	0.09 (< 0.01–1.57)	0.099
Inter-muscle fat fraction	0.01 (< 0.01– > 1000)	0.615
Skeletal muscle area (SM area)	**0.987 (0.975–0.999)**	**0.043**
Fat tissue in muscle area	1.02 (0.91–1.15)	0.679
Visceral adipose tissue area (VAT area)	**1.004 (1.002–1.007)**	**0.001**

Multivariable Cox regression analysis. Hazard ratios are presented with 95% confidence interval.

Significant values are in bold.

### Impact of VAT area and SM area on baseline characteristics and clinical outcome

**Figure 3:**
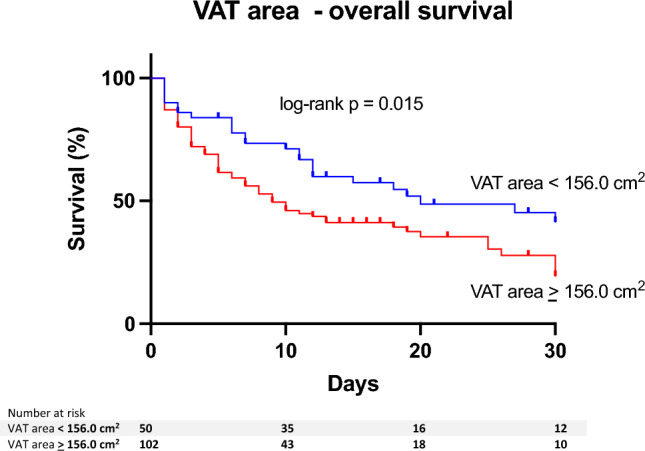
Kaplan–Meier curves illustrating 30-day survival of the entire study population (n = 152) stratified by VAT area. The cohort was divided using a cut-off value for VAT area (≷ 156.0 cm^2^). On log-rank test, 30-day survival significantly decreased with increasing VAT area (*P* = 0.015). HU = Hounsfield Units, VAT = visceral adipose tissue.

**Figure 4:**
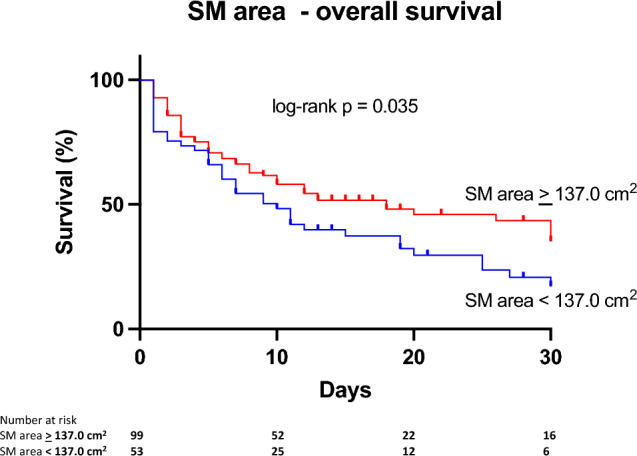
Kaplan–Meier curves illustrating 30-day survival of the entire study population (n = 152) stratified by SM area. The cohort was divided using a cut-off value for SM area (≷ 137 cm^2^). On log-rank test, 30-day survival significantly decreased with increasing SM area (*P* = 0.035). HU = Hounsfield Units, SM = skeletal muscle.

**Figure 5:**
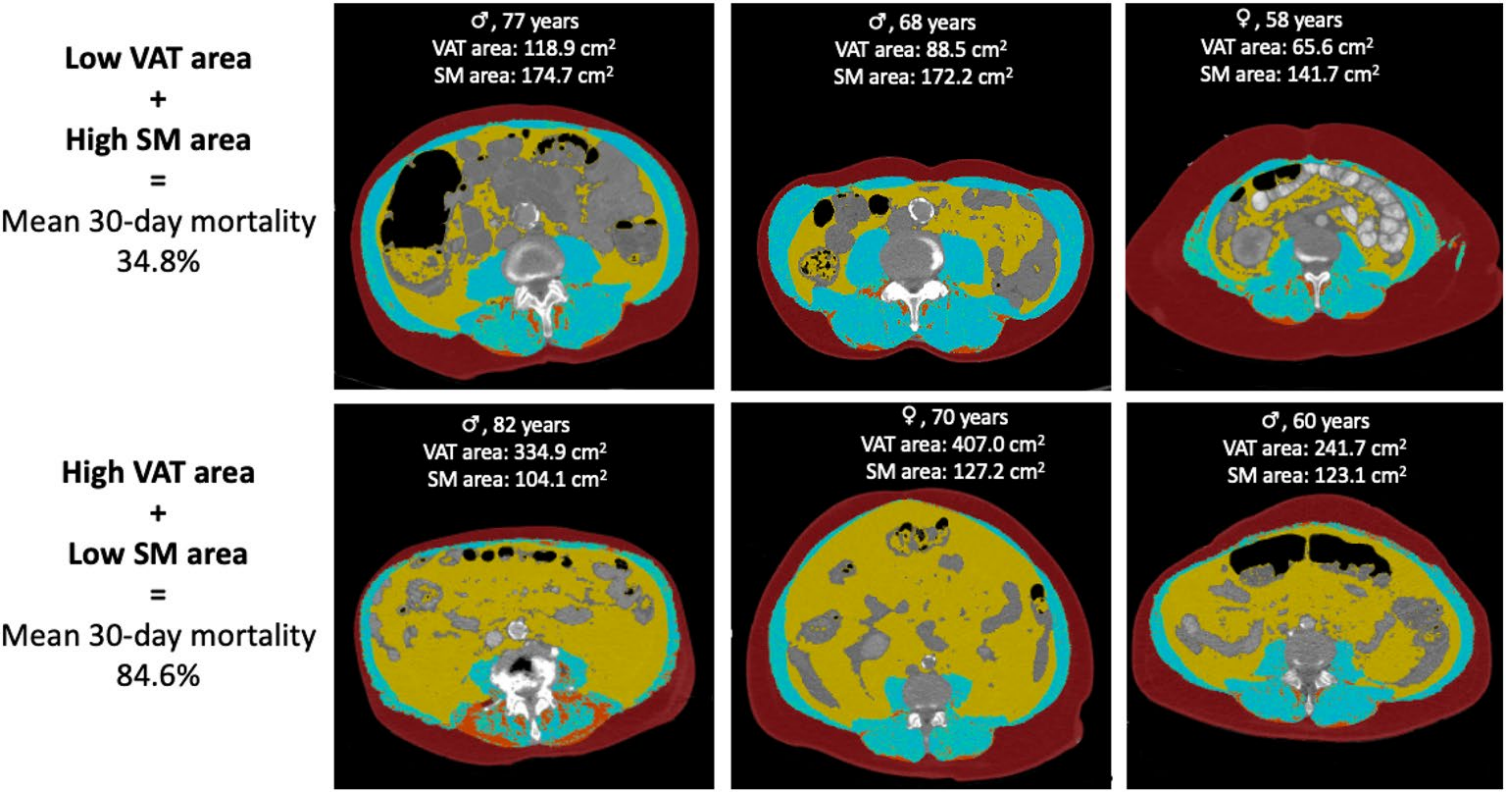
The images show exemplary male and female patients of different ages with low VAT area and high SM area, respectively, and with high VAT area and low SM area. Areas of visceral adipose tissue (yellow) and abdominal skeletal muscle (blue) were assessed within these cross-sections based on densitometric thresholds. High VAT area and low SM area were associated with higher 30-day mortality. SM = skeletal muscle, VAT = visceral adipose tissue.

We next divided the cohort using cut-off values for VAT area (≷ 156cm^2^) and SM area (≷ 137 cm^2^). Patients with a higher VAT area had a shorter overall survival time (OS 30 [7–30] vs. 10 [3–30] days, *p* = 0.045; Kaplan–Meier log-rank *p* = 0.015, Fig. 3). In contrast, patients with a higher SM area showed longer OS (10 [2.5–30] vs. 30 [5–30], *p* = 0.021; Kaplan–Meier log-rank *p* = 0.035, Fig. 4). Exemplary images of patients with high SM area and low VAT area, respectively, and of patients with low SM area and high VAT area are provided in Fig. 5.

Patients with higher VAT area were more likely to be male (82.0% vs. 58.3%, *p* = 0.004), had higher baseline creatinine (1.7 [1.3–2.3] mg/dl vs. 1.2 [1.0–1.6] mg/dl, *p* = 0.001), higher white blood cell count and presented with more severe metabolic acidosis as shown by Base excess and pH (Supplemental Table 2). Patients with higher SM area were also more likely to be male (90.6% vs. 44.2%, *p* < 0.001) and were younger (65.5 ± 13.3 years vs. 74.5 ± 10.5 years, *p* < 0.001). Moreover, a higher SM area was associated with lower c-reactive protein levels (9.0 [4.0–43.6] mg/l vs. 39.7 [11.4–133.9] mg/l, *p* = 0.006) and with higher hemoglobin levels (12.0 ± 2.9 g/dl vs. 10.9 ± 2.2 g/dl, *p* = 0.013; Supplemental Table 3). Finally, for both VAT area and SM area, we did not observe significant differences in the incidence of key interventions (Supplemental Table 4), including mechanical ventilation (VAT area: 68.6% vs. 64.0%, *p* = 0.586; SM area: 68.7% vs. 64.2%, *p* = 0.591), coronary angiography (VAT area: 79.4% vs. 78.0%, *p* = 0.999; SM area: 81.8% vs. 71.7%, *p* = 0.155), renal replacement therapy (VAT area: 39.2% vs. 28.0%, *p* = 0.208; SM area: 35.4% vs. 35.8%, *p* = 0.999), and the utilization of mechanical circulatory support (VAT area: 11.7% vs. 10.0%, *p* = 0.999; SM area: 11.1% vs. 11.3%, *p* = 0.999).

## Discussion

In our study, we investigated the predictive value of CT-derived parameters of fat and muscle tissue composition in patients presenting with cardiogenic shock. A higher VAT area and a lower SM area were associated with 30-day mortality, independent of already established clinical prognosticators, such as patient age or laboratory values. These two CT-derived parameters do not replicate prognostic information already available from clinical variables and therefore may have the potential to become valuable new tools for risk stratification in patients with CS.

Despite advances in medical therapy, CS is still associated with high morbidity and mortality, as well as an oftentimes uncertain prognosis^3^. Early identification of patients who could benefit most from more aggressive and invasive methods is of great importance for optimal patient allocation and to guide treatment strategy^24,25^. Currently, there are numerous risk scores available to assist in risk stratification and determination of an appropriate therapeutic approach. However, comparative studies report limited prognostic accuracy of existing CS risk scores^24^. In a meta-study, Sanjog et al. examined currently published CS risk scores regarding their predictive value and their applicability in clinical routine^2^. According to this study, lack of applicability to different patient populations is a major limitation of current CS risk scores. Most CS risk scores were developed and validated specifically for patients with CS second to acute myocardial infarction. These risk scores show reduced discriminatory performance when applied to patients with non-ischemic etiology of CS^25^. Although recent attempts have been made to analyze other CS etiologies, to date there are no accurate and validated CS risk scores that can be applied to all CS populations^2^.

Furthermore, many CS risk scores require a variety of clinical variables, laboratory results and comorbidities^2^. The inclusion of these multitude of different variables impairs implementation in everyday practice due to difficult and time-consuming calculations.

Knowing these limitations, in the present study we included an all-comers cohort of patients hospitalized with cardiogenic shock regardless of cause. Segmentation and analysis of abdominal adipose tissue and muscle tissue characteristics were performed fully automatically using a high-precision deep learning segmentation tool for connective tissue compartments based on previous diagnostic CT data^18^. Deep Learning has the potential to automate objective and reliable analysis of body composition parameters that can be opportunistically performed in parallel with image acquisition, specifically by end-to-end automation^19,26^. These conditions facilitate a potential rapid and uncompromised application of CT-derived biomarkers in clinical practice to assist in determining the appropriate therapeutic approach.

Recently, there has been a growing interest in the opportunistic assessment of fat and muscle composition based on cross-sectional images, as they have been shown to provide valuable prognostic insights for patients with various malignant conditions and cardiometabolic disorders^7,8,13,14^. Prior research hints at the significance of not just pure muscle mass but also its composition as a measure of muscle quality, particularly in patients undergoing interventional treatments^12,23^. Regarding adipose tissue, it has been demonstrated that, in addition to quantity, density changes associated with metabolic processes have also proven to be of prognostic significance^6,20^. Therefore, we made the decision to explore not only fat and muscle area but also the composition of fat and muscle in our patient cohort. In our study, SM area and VAT area emerged as independent predictors of survival. In alignment with numerous preceding studies, the assessment of SM and VAT area appears to provide pertinent prognostic information regarding survival duration that extend beyond what established baseline data and anthropometric characteristics capture^12,23^. Consequently, these measurements could potentially serve as straightforward point-of-care instruments for evaluating clinical status based on emergency diagnostic CT scans.

In our study, the BMI value was only available for 51 out of 152 patients. In this context, it is of utmost importance to consider that routine assessments, like measuring BMI, are often unfeasible in emergency rooms and intensive care units due to the critical condition of patients and time constraints. It is crucial to note that in most cases, BMI measurements were obtained only sporadically upon the patient's arrival in the ER and were typically not available for primary clinical decision-making. In our subanalysis, BMI also exhibited no significant associations with 30-day survival following cardiogenic shock. These findings align with numerous previously published studies, in which BMI, unlike VAT and SAT, either demonstrated no prognostic value for the outcomes of various cardiovascular and malignant diseases^27^ or where the significant additional prognostic value of the body composition parameters measured in our study persisted after adjustment for BMI^8,28,29^.

Currently, CT is not routinely indicated as initial imaging in all patients presenting with cardiogenic shock. However, certain clinical scenarios exist in which CT imaging is essential in these patients, such as aortic dissection, pulmonary embolism, or internal bleeding^15,16^. For these patients, additional evaluation of body composition parameters from already existing imaging data could potentially facilitate finding an appropriate therapeutic approach. Recent studies recommend the routine acquisition of CT images for patients admitted with cardiac arrest, as these may help to improve prognosis^30^.

There are some limitations to our study, such as its retrospective study design and the restricted sample size of patients. Moreover, we have no data available regarding long-term outcome and all patients were recruited from a single center. As mentioned above, the study population was heterogenous regarding CS etiology and disease progression. Moreover, only patients with suitable CT data for body composition analysis were enrolled into the study, thus selection bias cannot be excluded. It is important to consider that our chosen cut-off values should be viewed solely as exploratory and hypothesis-generating. Clinically applicable cut-off values in larger cohorts need to be determined in future studies. However, the independent association between SM area, VAT area and patient outcomes within this heterogeneous cohort underscores the potential utility, robustness and broad applicability of CT-derived body composition analysis.

In this study we propose the use of VAT area and SM area, representing objective measures of patient clinical status, as promising imaging biomarkers for the outcome of patients with CS. The potentially outstanding value of VAT area and SM area is underscored by the fact that, unlike most CS risk scores, they can be rapidly and easily determined using available diagnostic imaging. Future prospective studies are required to further investigate these findings.

### Supplementary Information

Supplementary Table 1.

Supplementary Table 2.

Supplementary Table 3.

Supplementary Table 4.

## Data Availability

Data generated or analyzed during the study are available from the corresponding author by reasonable request.
