# Supplementary material for: Opportunistic CT-derived analysis of fat and muscle tissue composition predicts mortality in patients with cardiogenic shock
Source: Sci Rep. 2023 Dec 15;13:22293. doi: 10.1038/s41598-023-49454-x (PMC10724270; doi:10.1038/s41598-023-49454-x)
Supplement: Supplementary file 1 — Supplementary Table 1. [file 41598_2023_49454_MOESM1_ESM.docx]

**Supplemental Table S1.** Clinical and anthropometric baseline characteristics of the study population (n=152) presenting with cardiogenic shock (CS) stratified by 30-day survival.

| Variables | Survivors  n = 62 | Non-Survivors  n = 90 | p value |
| --- | --- | --- | --- |
| Age (years) | **62.3 ± 13.8** | **72.9 ± 10.6** | **<.001** |
| Male sex | 47 (75.8 %) | 63 (70.0 %) | 0.849 |
| Acute myocardial infarction | 35 (56.5 %) | 42 (46.7 %) | 0.252 |
| Cardiac arrest (%) | **38 (61.3 %)** | **63 (70.0 %)** | **0.019** |
| Lactate (mmol/l) | 5.4 (2.3-8.6) | 7.1 (3.1-11.5) | 0.083 |
| pH | 7.30 (7.08-7.38) | 7.25 (7.12-7.35) | 0.272 |
| Base excess | -7.4 (-11.7- -3.1) | -8.6 (-12.6 - -5.75) | 0.209 |
| Creatinine (mg/dl) | **1.36 (1.09-1.80)** | **1.75 (1.28-2.52)** | **0.002** |
| Hemoglobin (g/dl) | **12.3 ± 2.9** | **11.1 ± 2.5** | **0.015** |
| White blood cell count (10^3^/µl) | 13.8 (10.3-18.4) | 14.1 (10.2-20.7) | 0.560 |
| Platelet count (10^3^/µl) | 201 (142-303) | 207 (159-302) | 0.853 |
| C-reactive protein (mg/l) | **9.4 (3.2-53.5)** | **25.1 (6.9-131)** | **0.029** |
| Overall survival (days) | **30 (30-30)** | **5 (2-11)** | **<.001** |

Data are presented as n (%), mean ± standard deviation if normally distributed and as median (interquartile range) if not normally distributed. Laboratory values at admission are shown.
