# Supplementary material for: Opportunistic CT-derived analysis of fat and muscle tissue composition predicts mortality in patients with cardiogenic shock
Source: Sci Rep. 2023 Dec 15;13:22293. doi: 10.1038/s41598-023-49454-x (PMC10724270; doi:10.1038/s41598-023-49454-x)
Supplement: Supplementary file 2 — Supplementary Table 2. [file 41598_2023_49454_MOESM2_ESM.docx]

**Supplemental Table S2.** Clinical and anthropometric baseline characteristics of the study population (n=152) presenting with cardiogenic shock (CS) stratified by visceral adipose tissue (VAT) area (≷156 cm^2^).

| Variables | VAT area < 156 cm^2^  n = 50 | VAT area > 156 cm^2^  n = 102 | p value |
| --- | --- | --- | --- |
| Age (years) | 68.9 ± 15.4 | 68.5 ± 11.9 | 0.850 |
| Male sex | **28 (58.3 %)** | **82 (82.0 %)** | **0.004** |
| Acute myocardial infarction | 24 (48.0 %) | 53 (52.0 %) | 0.731 |
| Cardiac arrest | 32 (64.0 %) | 69 (67.6 %) | 0.731 |
| Lactate (mmol/l) | 6.3 (2.9-9.7) | 7.6 (2.9-10.5) | 0.543 |
| pH | **7.36 (7.13-7.40)** | **7.24 (7.09-7.34)** | **0.017** |
| Base excess | **-6.2 (-10.2- -2.7)** | **-9.6 (-13.3- -4.9)** | **0.030** |
| Creatinine (mg/dl) | **1.2 (1.0-1.6)** | **1.7 (1.3-2.3)** | **0.001** |
| Hemoglobin (g/dl) | 11.3 ± 2.4 | 11.8 ± 2.8 | 0.317 |
| White blood cell count (10^3^/µl) | **11.6 (9.3-17.0)** | **14.3 (10.6-20.9)** | **0.046** |
| Platelet count (10^3^/µl) | 194 (141-269) | 205 (153-309) | 0.223 |
| C-reactive protein (mg/l) | 14.1 (6.2-101.0) | 16.5 (4.9-58.0) | 0.484 |
| Overall survival (days) | **30 (7-30)** | **10 (3-30)** | **0.045** |

Data are presented as n (%), mean ± standard deviation if normally distributed and as median (interquartile range) if not normally distributed. Laboratory values at admission are shown.
