# Supplementary material for: Opportunistic CT-derived analysis of fat and muscle tissue composition predicts mortality in patients with cardiogenic shock
Source: Sci Rep. 2023 Dec 15;13:22293. doi: 10.1038/s41598-023-49454-x (PMC10724270; doi:10.1038/s41598-023-49454-x)
Supplement: Supplementary file 3 — Supplementary Table 3. [file 41598_2023_49454_MOESM3_ESM.docx]

**Supplemental Table S3.** Clinical and anthropometric baseline characteristics of the study population (n=152) presenting with cardiogenic shock (CS) stratified by skeletal muscle (SM) area (≷137 cm^2^).

| Variables | SM area < 137 cm^2^  n = 53 | SM area > 137 cm^2^  n = 99 | p value |
| --- | --- | --- | --- |
| Age (years) | **74.5 ± 10.5** | **65.5 ± 13.3** | **<.001** |
| Male sex | **23 (44.2 %)** | **87 (90.6 %)** | **<.001** |
| Acute myocardial infarction | 27 (50.9 %) | 50 (50.5 %) | 0.959 |
| Cardiac arrest (%) | 34 (64.2 %) | 67 (67.7 %) | 0.768 |
| Lactate (mmol/l) | 6.7 (4.5-11.0) | 6.2 (2.2-9.4) | 0.132 |
| pH | 7.28 (7.09-3.37) | 7.25 (7.13-7.37) | 0.828 |
| Base excess | -8.2 (-12.3- -5.9) | -7.6 (-12.9- -3.8) | 0.365 |
| Creatinine (mg/dl) | 1.52 (1.05-2.40) | 1.60 (1.20-2.10) | 0.686 |
| Hemoglobin (g/dl) | **10.9 ± 2.2** | **12.0 ± 2.9** | **0.013** |
| White blood cell count (10^3^/µl) | 15.0 (10.6-21.0) | 13.3 (10.1-18.5) | 0.287 |
| Platelet count (10^3^/µl) | 220 (156-325) | 198 (146-295) | 0.292 |
| C-reactive protein (mg/l) | **39.7 (11.4-133.9)** | **9.0 (4.0-43.6)** | **0.006** |
| Overall survival (days) | **10 (2.5-30)** | **30 (5-30)** | **0.021** |

Data are presented as n (%), mean ± standard deviation if normally distributed and as median (interquartile range) if not normally distributed. Laboratory values at admission are shown.
