# Supplementary material for: Opportunistic CT-derived analysis of fat and muscle tissue composition predicts mortality in patients with cardiogenic shock
Source: Sci Rep. 2023 Dec 15;13:22293. doi: 10.1038/s41598-023-49454-x (PMC10724270; doi:10.1038/s41598-023-49454-x)
Supplement: Supplementary file 4 — Supplementary Table 4. [file 41598_2023_49454_MOESM4_ESM.docx]

**Supplemental Table S4.** Description of key interventions and therapies stratified by visceral adipose tissue (VAT) area (≷156 cm^2^) and skeletal muscle (SM) area (≷137 cm^2^).

| Variables | VAT area < 156 cm^2^  n = 50 | VAT area > 156 cm^2^  n = 102 | p value | SM area < 137 cm^2^  n = 53 | SM area > 137 cm^2^  n = 99 | p value |
| --- | --- | --- | --- | --- | --- | --- |
| Mechanical ventilation | 32 (64.0 %) | 70 (68.6 %) | 0.586 | 34 (64.2 %) | 68 (68.7 %) | 0.591 |
| Coronary angiography | 39 (78.0 %) | 80 (79.4 %) | 1.000 | 38 (71.7 %) | 81 (81.8 %) | 0.155 |
| Renal replacement therapy | 14 (28.0 %) | 40 (39.2 %) | 0.208 | 19 (35.8 %) | 35 (35.4 %) | 1.000 |
| Mechanical circulatory support | 5 (10.0 %) | 12 (11.7 %) | 1.000 | 6 (11.3 %) | 11 (11.1 %) | 1.000 |

Data are presented as n (%).
